# Sympathetic innervation of human and porcine spleens: implications for between species variation in function

**DOI:** 10.1186/s42234-022-00102-1

**Published:** 2022-12-19

**Authors:** Logan G. Kirkland, Chloe G. Garbe, Joseph Hadaya, Paul V. Benson, Brant M. Wagener, Sanjin Tankovic, Donald B. Hoover

**Affiliations:** 1 Department of Biomedical Sciences, Quillen College of Medicine, East Tennessee State University, Johnson City, TN 37614 USA; 2 UCLA Cardiac Arrhythmia Center and Neurocardiology Research Program of Excellence, David Geffen School of Medicine at UCLA, Los Angeles, CA 90095 USA; 3 Molecular, Cellular, and Integrative Physiology Program, University of California, Los Angeles, Los Angeles, CA USA; 4 Department of Pathology, The University of Alabama at Birmingham, Heersink School of Medicine, Birmingham, AL 35249 USA; 5 Department of Anesthesiology and Perioperative Medicine, The University of Alabama at Birmingham, Heersink School of Medicine, Birmingham, AL 35249 USA; 6 Department of Biomedical Sciences, Quillen College of Medicine and Center of Excellence in Inflammation, Infectious Disease and Immunity, East Tennessee State University, Johnson City, TN 37614 USA

**Keywords:** Spleen, Noradrenergic innervation, Immunohistochemistry, Tyrosine hydroxylase, Neuropeptide Y, Leukocytes, T cells, Arteries, Cholinergic anti-inflammatory pathway

## Abstract

**Background:**

The vagus nerve affects innate immune responses by activating spleen-projecting sympathetic neurons, which modulate leukocyte function. Recent basic and clinical research investigating vagus nerve stimulation to engage the cholinergic anti-inflammatory pathway (CAP) has shown promising therapeutic results for a variety of inflammatory diseases. Abundant sympathetic innervation occurs in rodent spleens, and use of these species has dominated mechanistic research investigating the CAP. However, previous neuroanatomical studies of human spleen found a more restricted pattern of innervation compared to rodents. Therefore, our primary goal was to establish the full extent of sympathetic innervation of human spleens using donor tissue with the shortest procurement to fixation time. Parallel studies of porcine spleen, a large animal model, were performed as a positive control and for comparison.

**Methods:**

Human and porcine spleen tissue were fixed immediately after harvest and prepared for immunohistochemistry. Human heart and porcine spleen were stained in conjunction as positive controls. Several immunohistochemical protocols were compared for best results. Tissue was stained for tyrosine hydroxylase (TH), a noradrenergic marker, using VIP purple chromogen. Consecutive tissue slices were stained for neuropeptide Y (NPY), which often co-localizes with TH, or double-labelled for TH and CD3, a T cell marker. High-magnification images and full scans of the tissue were obtained and analyzed for qualitative differences between species.

**Results:**

TH had dominant perivascular localization in human spleen, with negligible innervation of parenchyma, but such nerves were abundant throughout ventricular myocardium. In marked contrast, noradrenergic innervation was abundant in all regions of porcine spleen, with red pulp having more nerves than white pulp. NPY stain results were consistent with this pattern. In human spleen, noradrenergic nerves only ran close to T cells at the boundary of the periarterial lymphatic sheath and arteries. In porcine spleen, noradrenergic nerves were closely associated with T cells in both white and red pulp as well as other leukocytes in red pulp.

**Conclusion:**

Sympathetic innervation of the spleen varies between species in both distribution and abundance, with humans and pigs being at opposite extremes. This has important implications for sympathetic regulation of neuroimmune interactions in the spleen of different species and focused targeting of the CAP in humans.

**Supplementary Information:**

The online version contains supplementary material available at 10.1186/s42234-022-00102-1.

## Background

Efferent neuronal regulation of the immune system occurs through the sympathetic nervous system, which innervates primary and secondary lymphoid tissues including bone marrow (BM), thymus, and the spleen (Bellinger and Lorton, 2014, Elenkov et al., 2000, Jung et al., 2017, Madden, 2017, Padro and Sanders, 2014). Norepinephrine released from sympathetic nerves in each of these tissues can affect various aspects of immune system function such as differentiation and proliferation of BM stem cells, trafficking of cells from the BM, spleen, and lymph nodes, and modulation of innate and adaptive immune responses. These actions are mediated through direct stimulation of specific α- and β-adrenergic receptor subtypes expressed by BM stem cells and specific populations of leukocytes (Bellinger and Lorton, 2014, Elenkov et al., 2000, Jung et al., 2017, Madden, 2017, Padro and Sanders, 2014). Direct sympathetic actions on leukocytes do not occur through classical synapses with postjunctional specializations but rather by release of neurotransmitter from varicose nerve fibers located at variable distances (20 nm to microns) from the target cells (Elenkov et al., 2000, Murray et al., 2017). Indirect sympathetic influence on lymphoid tissues can also occur through activation of the adrenal medulla and release of catecholamines (predominantly epinephrine) into the circulation (Elenkov et al., 2000).

​ Direct sympathetic modulation of leukocytes in the spleen has become a topic of intense interest in recent years due to the discovery of the cholinergic anti-inflammatory pathway (CAP), which provides a novel approach for activating anti-inflammatory mechanisms in the spleen (Falvey et al., 2022, Hoover, 2017, Kelly et al., 2022, Pavlov and Tracey, 2022). This system is unique in having two unexpected cholinergic elements: 1) preganglionic vagal cholinergic input to sympathetic neurons in the celiac/superior mesenteric ganglia and 2) cholinergic T cells, which have a crucial role in non-neuronal release of acetylcholine (ACh) in the spleen. Stimulation of vagal efferent fibers activates spleen projecting sympathetic neurons in the celiac/superior mesenteric ganglia (Hoover, 2017, Kressel et al., 2020, Lehner et al., 2019, Murray et al., 2019, Murray et al., 2021, Pavlov and Tracey, 2022). These nerve fibers travel to the spleen in the splenic nerve, which enters the organ at the hilum with the splenic artery (Bellinger et al., 1989, Felten et al., 1985, Sokal et al., 2021). Noradrenergic nerve fibers then travel along the arterial vasculature and ultimately send projections into the white pulp region of the parenchyma. Activation of these fibers by vagal stimulation induces release of norepinephrine (NE) from varicosities, and stimulation of β_2_-adrenergic receptors on a subset of cholinergic CD4 + T cells by NE causes local release of ACh (Rosas-Ballina et al., 2011, Sokal et al., 2021, Vida et al., 2011a, Vida et al., 2011b). Next, T cell derived ACh diffuses to macrophages located in the marginal zone and possibly other sites in the spleen, where it evokes an anti-inflammatory response through activation of α7 nicotinic ACh receptors (Hoover, 2017, Pavlov and Tracey, 2022, Wang et al., 2003). Specifically, α7 activation inhibits the production and release of proinflammatory cytokines such as tissue necrosis factor-α and interleukin-6.

Overwhelming evidence from preclinical studies has demonstrated the therapeutic potential of the CAP for treating a wide range of diseases (Falvey et al., 2022, Hoover, 2017, Pavlov and Tracey, 2019, Pavlov and Tracey, 2022). This success has naturally fostered translational studies aimed at applying vagal nerve stimulation (VNS) technology to chronic inflammatory diseases such as rheumatoid arthritis and inflammatory bowel disease (Pavlov and Tracey, 2022). Ongoing work to refine and improve technology for human therapeutic applications includes the use of non-invasive technology and the development of durable bioelectronic devices for direct stimulation of the vagus or smaller nerves, such as the splenic nerve (Donegà et al. 2021, Gupta et al., 2020, Pavlov and Tracey, 2022, Sokal et al., 2021). Despite the promising future for bioelectronic activation of the CAP, surprisingly little is known about its mechanisms within human spleen.

​ Several recent studies including ours have specifically evaluated noradrenergic innervation of the human spleen (Cleypool et al., 2021, Hoover et al., 2017, Verlinden et al., 2019), and all identified innervation of splenic arteries down to the level of central arteries and arterioles, which are surrounded by white pulp. Two of these studies reported a few noradrenergic nerve fibers making short excursions into surrounding white pulp (Cleypool et al., 2021, Hoover et al., 2017), and one reported that some noradrenergic fibers occurred near CD3 + T cells (Cleypool et al., 2021). However, none of them observed dense sympathetic innervation of the white pulp as reported for mice and rats.

Since the anatomical proximity of sympathetic nerves to leukocytes is assumed to be important for the anti-inflammatory mechanism elucidated in small animals, we have evaluated this issue using human spleen samples collected prospectively from organ donors. This approach enables rapid fixation of viable tissue and minimizes the potential for post-mortem changes. Various permutations of the immunohistochemical staining protocol for the noradrenergic marker, tyrosine hydroxylase (TH), were evaluated to assure maximum visualization of sympathetic nerves in samples collected from different regions of the spleen. Human heart and porcine spleen samples were evaluated as positive controls. Since neuropeptide Y (NPY) is a recognized co-transmitter in sympathetic nerves, we also evaluated the presence and localization of this marker in spleen samples. Double labeling methods were used to evaluate the proximity of TH + nerves to CD3 + T cells.

## Methods

### Tissue collection and preparation

Use of human tissue for this study was approved by the Institutional Review Board of the University of Alabama Birmingham (UAB) (Protocol# 300001087 and 300005723) and was conducted in accordance with the Declaration of Helsinki. Samples of human spleen were collected from six brain dead donors after organ procurement at the UAB Legacy of Hope. Demographic and patient history information for the donors is listed in Table 1.

**Table 1:** Demographics and patient history

Donor	Sex	Age	Race/Ethnicity	Cause of Death	Health History
001	M	33	Unknown/Non-Hispanic	MVA	N/A, significant chronic alcohol consumption
002	F	59	White/Non-Hispanic	SAH	HTN,CAD,COPD, prior CVA in the setting of SAH, methamphetamine use
003	F	46	White/Non-Hispanic	MI vs. SAH	Psoriatic arthritis, prior cardiac arrests, SAH, smoked 1 pack cigarettes/day
004	M	57	Unknown/Non-Hispanic	GSW to head	Hypercholesterolemia, smoked 2 packs cigarettes/day, significant chronic alcohol consumption
005	M	54	White/Non-Hispanic	Brain death	Hypertension, CAD, prior heart attack, untreated TIA, smoked 1 pack cigarettes/day, smoked marijuana daily, recreational powder cocaine use
006	M	30	White/Non-Hispanic	anoxic brain injury, MVA	Morbidly obese, history of IV heroin use, oral pain med abuse, and marijuana use, smoked 1 pack cigarettes/day

After removal of donor spleens, tissue was dissected into a 1–2 cm block of about 0.5 -1 cm thickness and was immediately fixed for 3–7 days in 10% neutral buffered formalin at room temperature before embedding in paraffin. Paraffin blocks were then shipped to East Tennessee State University (ETSU) for study. We also received paraffin sections of normal human left ventricle from UAB to use as a positive control for sympathetic nerve staining. Samples of porcine spleen (Yucatan mini-pig) were provided by UCLA for use as a positive control and to evaluate sympathetic innervation of spleen in another large species. Porcine spleens were collected at the end of terminal experiments approved by the UCLA Institutional Animal Care and Use Committee. These samples were dissected into blocks of about the same size as used for human spleen, fixed for 3–7 days in 10% neutral buffered formalin, and transferred to 70% ethanol for shipment to ETSU with ice packs. Porcine samples were embedded in paraffin at ETSU. Two spleens were collected from normal Yucatan mini-pigs and one more from a mini-pig at six weeks after myocardial infarction. Paraffin sections were cut at 5 μm thickness using a Leica RM2135 microtome and collected on SuperFrost® Plus slides (Fisher Scientific).

### Histology and immunohistochemistry

Representative sections from each human spleen were stained with hematoxylin & eosin (H&E) by standard methods to view cellular organization and morphology. Immunohistochemistry was used to stain for TH (noradrenergic marker) or NPY (co-transmitter in sympathetic nerves). Prior to staining, sections were deparaffinized, hydrated, and treated for antigen retrieval (either 1 mM ethylenediaminetetraacetic acid (EDTA), pH 8, 30 min at 92 °C or 1 mM citrate, pH 6.0, 30 min at 92 °C). Staining was done at room temperature using the Avidin-Biotin Complex (ABC) immunohistochemistry method (Rabbit ABC- horseradish peroxidase (HRP) Kit, PK-4001, Vector Labs). Briefly, slides were rinsed with phosphate buffered saline (PBS, pH 7.3), incubated for 10 min in PBS containing 0.4% Triton X-100 and 0.5% bovine serum albumin (BSA), treated for 15 min with 1.0% H_2_O_2_ in PBS, rinsed an additional time with PBS and incubated 10 min in PBS containing 0.4% Triton X-100 and 0.5% BSA. Slides were then placed in an incubation box and covered with blocking buffer (PBS containing 1% BSA, 0.4% Triton X-100, and normal goat serum). After 2 h, the blocking buffer was replaced with fresh blocking buffer containing the primary antibody (rabbit anti-TH, 1:500, Pel-Freeze P40101-150; rabbit anti-NPY, 1:1000, Immunostar 22,940) and incubated overnight at room temperature. Sections were washed with PBS and PBS containing 0.5% BSA followed by a two-hour incubation in biotinylated secondary antibody (1:200 dilution) from the kit. Slides were washed again before a 1.5-h incubation with the ABC reagent from the kit. Slides were next washed for 20 min in 50 mM Tris buffer (pH 7.6) before treatment for 1–10 min with the chromogen (Vector ImmPACT VIP Kit, SK4605) to visualize targets (purple reaction product). Slides were washed, dehydrated, and cover glasses were attached using Cytoseal XYL (Thermo Scientific Cat. No. 8312–4).

The TH and NPY stains were repeated using the Vector ImmPRESS Excel Amplified Polymer Kit for Anti-Rabbit IgG, Peroxidase (Vector Laboratories, Inc., USA, MP7601), following the manufacturer-recommended protocol. This kit uses a diaminobenzidine (DAB) chromogen (brown reaction product).

To examine the association of TH + nerves with CD3 + T cells, double labeling experiments were performed using the Vector ImmPRESS Duet Double Staining Polymer Kit (Vector, MP7714), with a modified version of the manufacturer-recommended protocol. The kit used 2.5% normal horse serum blocking buffer, and sections were incubated with a PBS solution containing 0.4% Triton X-100, 1% BSA, and two primary antibodies (rabbit anti-TH and mouse anti-CD3, 1:20, Abcam ab11089). The ImmPRESS Duet HRP/AP (alkaline phosphatase) Polymer Reagent included a mixture of HRP anti-rabbit IgG and AP anti-mouse IgG. The tissue was developed first with DAB EqV Substrate, yielding brown reaction product for TH. This was followed by washing and the addition of Red Substrate, yielding a magenta product for CD3.

### Microscopy and image analysis

Stained sections were viewed, and digital images collected using an Olympus BX41 microscope equipped with an Olympus DP74 digital camera and cellSens Dimension software (Olympus America Inc., Center Valley, PA). Additionally, digital images of full sections were collected for a subset of experiments using a Leica Biosystems Aperio CS Console (Leica Biosystems Imaging, Inc., CA, USA). Magnification for all full scans was set to 20X, and all controls were set to the manufacturer’s default settings.

To quantify the extent of vasculature innervation by noradrenergic nerves within human spleens, total number of blood vessels within two standardized areas of 26 mm^2^, selected for consistency across samples of different sizes, were manually counted in three regions (hilum, pole, and outer wall) of tissue stained with H&E using Aperio ImageScope 12.4.6. (Leica Biosystems Imaging, Inc., CA, USA). This was repeated in the same regions on consecutive slides stained for TH, counting only the number of innervated blood vessels within the region of interest. These numbers were used to estimate the percentage of innervated blood vessels in each region. Statistical analysis was done using Prism version 8.4.3 (GraphPad Software, San Diego, CA). Values obtained for different regions of spleen were compared using repeated measures analysis of variance (*P* < 0.05) and pairwise comparisons conducted using Tukey’s multiple comparisons test.

## Results

### Immunohistochemical protocol and antigen retrieval method optimization

**Fig. 1:**
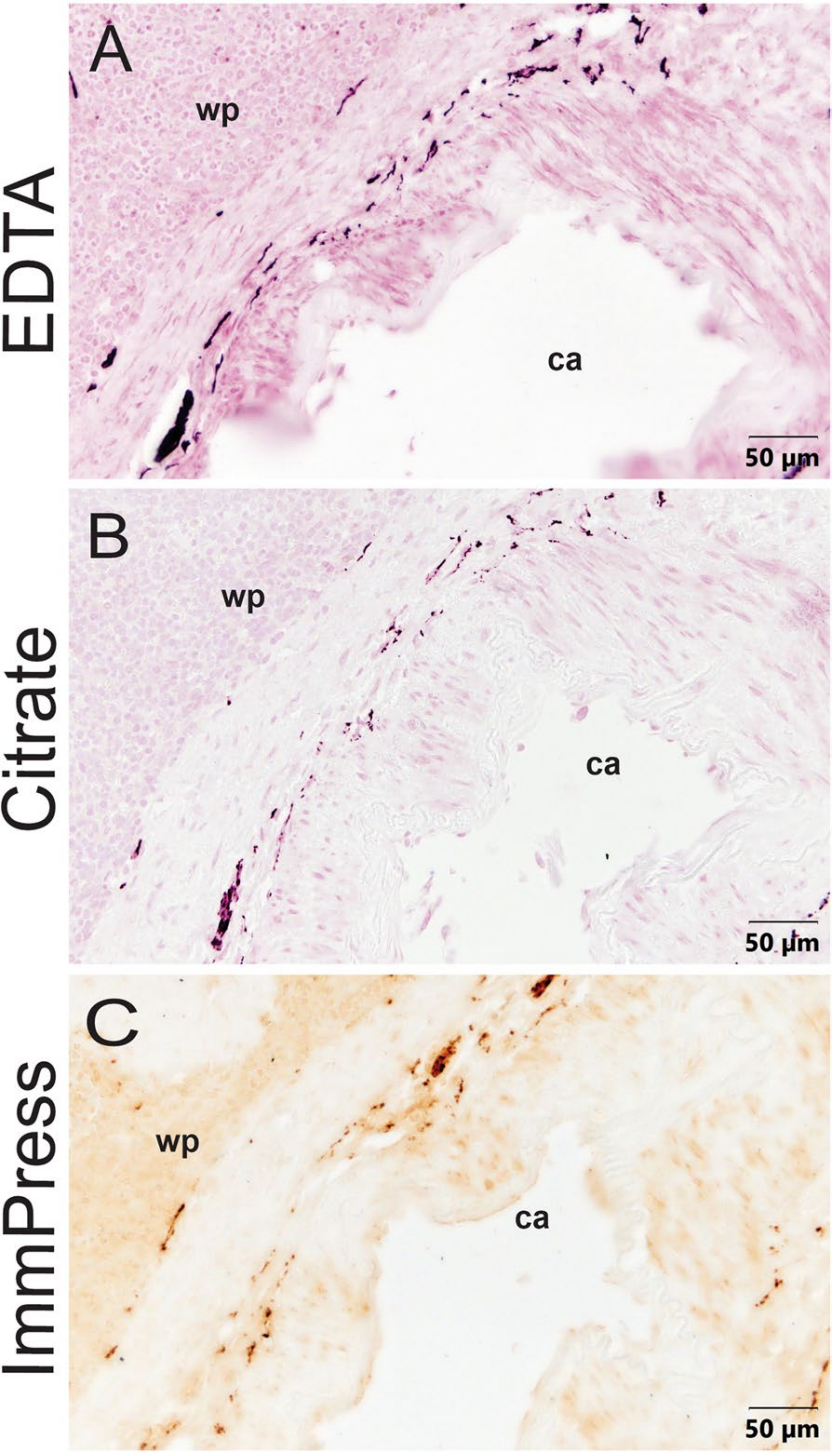
Comparison of different IHC antigen retrieval buffers and chromogens used to stain spleen tissue. Images were taken from three consecutive sections of spleen from a 57-year-old male (donor 004) and stained for TH. Images show white pulp (wp) and a central artery (ca.). **A** VIP purple chromogen using EDTA antigen retrieval showed greater intensity of nerve staining as well as higher degree of tissue degradation. **B** VIP purple chromogen using citrate buffer antigen retrieval better preserved the adventitia of blood vessels when compared to EDTA antigen retrieval. Additionally, spleen sections stained for TH using citrate antigen retrieval yielded less background staining than the other two methods. **C** ImmPRESS DAB chromogen using citrate buffer antigen retrieval showed fewer sympathetic nerves than adjacent sections

Most previous studies evaluating sympathetic innervation of the human spleen used samples collected during autopsy and fixed at unspecified times after death. Since there is the potential for loss of some immunostaining during this interval, we circumvented this issue by using spleen samples collected after organ retrieval and fixed immediately. To be certain that our immunostaining for TH revealed complete noradrenergic innervation of the human spleen, we evaluated sections from the same samples from each donor using different permutations of the immunohistochemical protocol and different antigen retrieval methods. Staining with the ABC Elite kit and VIP chromogen yielded similar localization of noradrenergic nerves after citrate or EDTA antigen retrieval (Fig. 1 A and B). Although staining was more intense after EDTA retrieval, this approach caused some regional damage to the spleen sections. Previous studies observed that the higher pH of EDTA antigen retrieval solution typically leads to faster and more thorough antigen retrieval but is also more likely to cause some tissue loss (Krenacs et al., 2010). Staining with the Vector ImmPRESS kit and DAB chromogen (citrate retrieval) was less intense, although the overall pattern of innervation was similar (Fig. 1 C).

### Noradrenergic innervation of the human spleen is restricted primarily to arterial vasculature

**Fig. 2:**
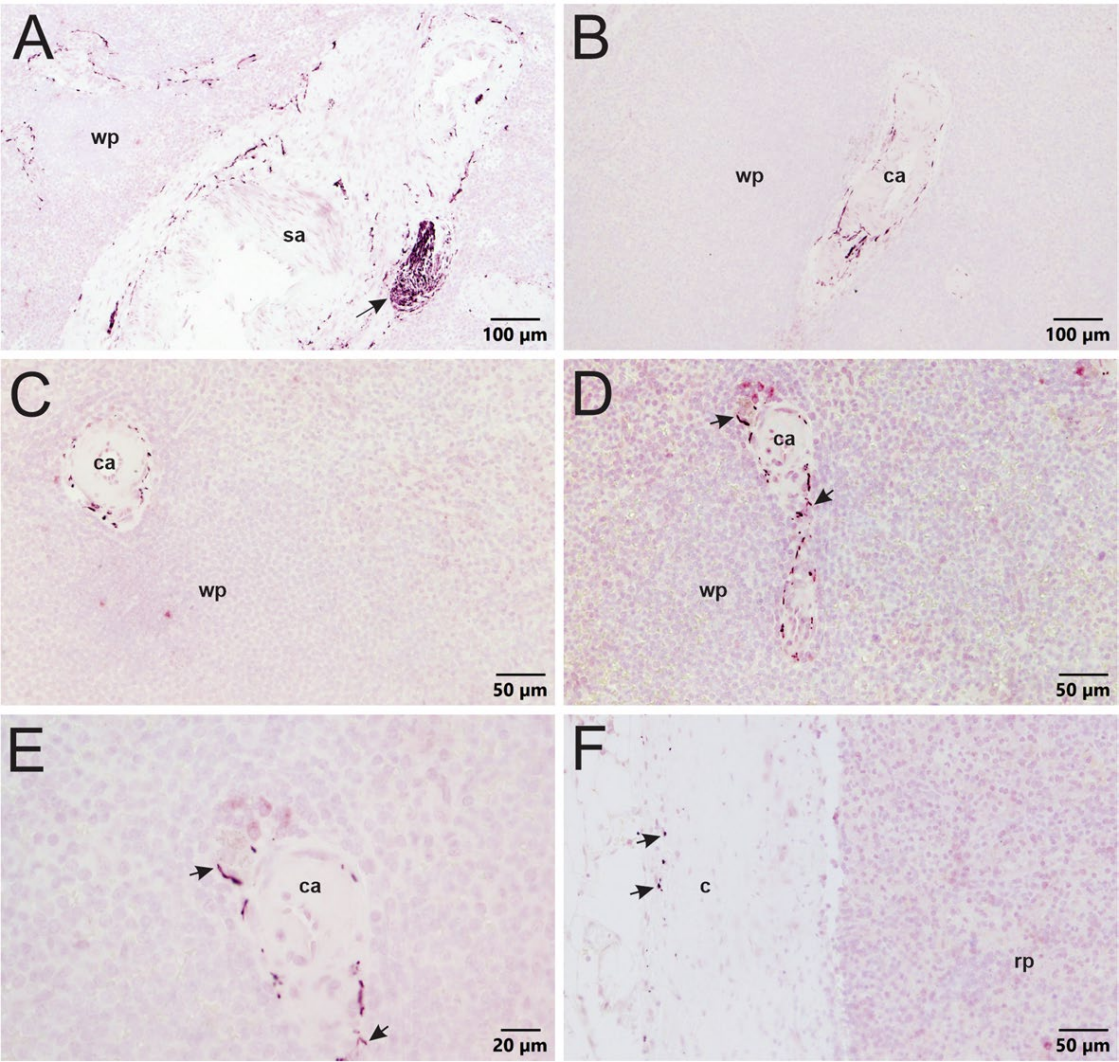
TH + innervation of normal human spleens using VIP chromogen and citrate buffer antigen retrieval. Sympathetic nerves were located primarily in the adventitia surrounding blood vessels and only rarely extended into the white pulp (wp). **A** A longitudinal cut showing the splenic artery (sa) and sympathetic innervation within the surrounding adventitia. Arrow indicates a nerve bundle. **B** Sympathetic innervation of a moderate-sized central arteriole (ca.) within white pulp. **C** Higher magnification image of a smaller arteriole surrounded by nerves. **D** Sympathetic nerves run along a blood vessel cut longitudinally. Arrows indicate TH + nerves extending a short distance into the surrounding white pulp. **E** Higher magnification of same arteriole in panel D showing nerves (arrows) protruding into the white pulp. **F** Arrows indicate TH + nerves cut in cross-section within the capsule (c). Note the absence of innervation in the red pulp (rp) to the right

**Fig. 3:**
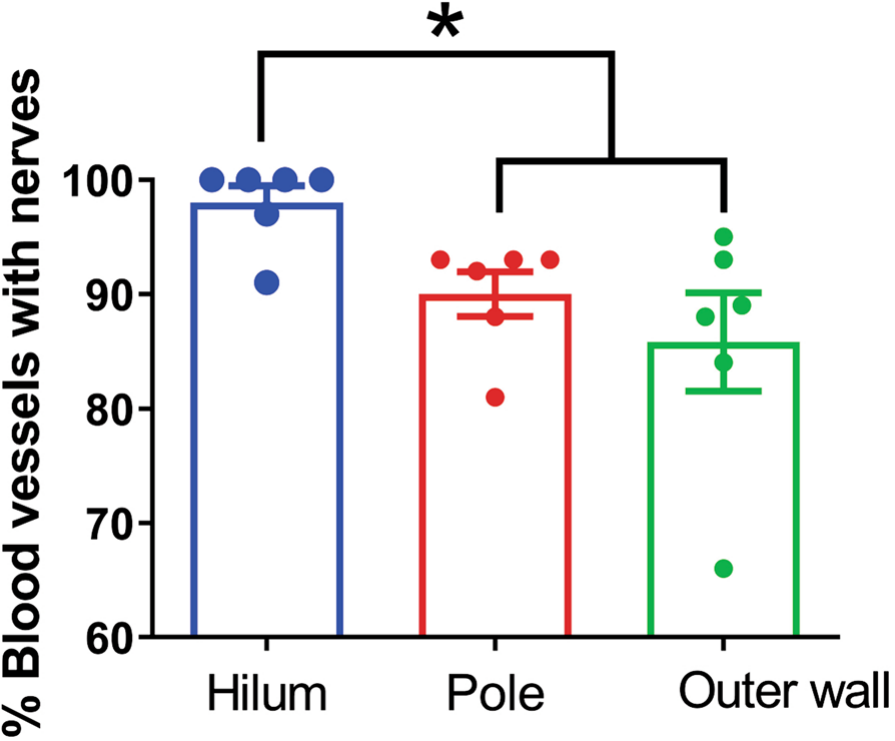
Quantification of blood vessel innervation in three regions of human spleen. Values are the mean ± SEM, *n* = 6. *Indicates a significant difference from all other groups (*P* = 0.045 for repeated measures analysis of variance; Tukey’s multiple comparison test)

Three regions of spleen from six human donors were evaluated for the presence and distribution of noradrenergic nerves. With the notable exception of human spleen 002, TH + nerves occurred primarily in the wall of arteries ranging in size from the splenic artery to arterioles within the white pulp (Fig. 2, Additional file 1). Larger arteries often contained prominent nerve bundles, while single nerves fibers were dominant in smaller arteries. Occasionally, small TH + nerves fibers penetrated a short distance into the surrounding parenchyma (Fig. 2D and E), but they never ramified throughout the white pulp as reported for rodents (Bellinger et al., 1989, Felten et al., 1985, Murray et al., 2017). Quantitative analysis of the incidence of sympathetic nerves in arteries and arterioles showed that a vast majority of these blood vessels contained TH + nerves (Fig. 3). Innervation at other sites within the spleen was rare, with a few nerves occurring in or directly beneath the capsule in some spleens (Fig. 2 F).

**Fig. 4:**
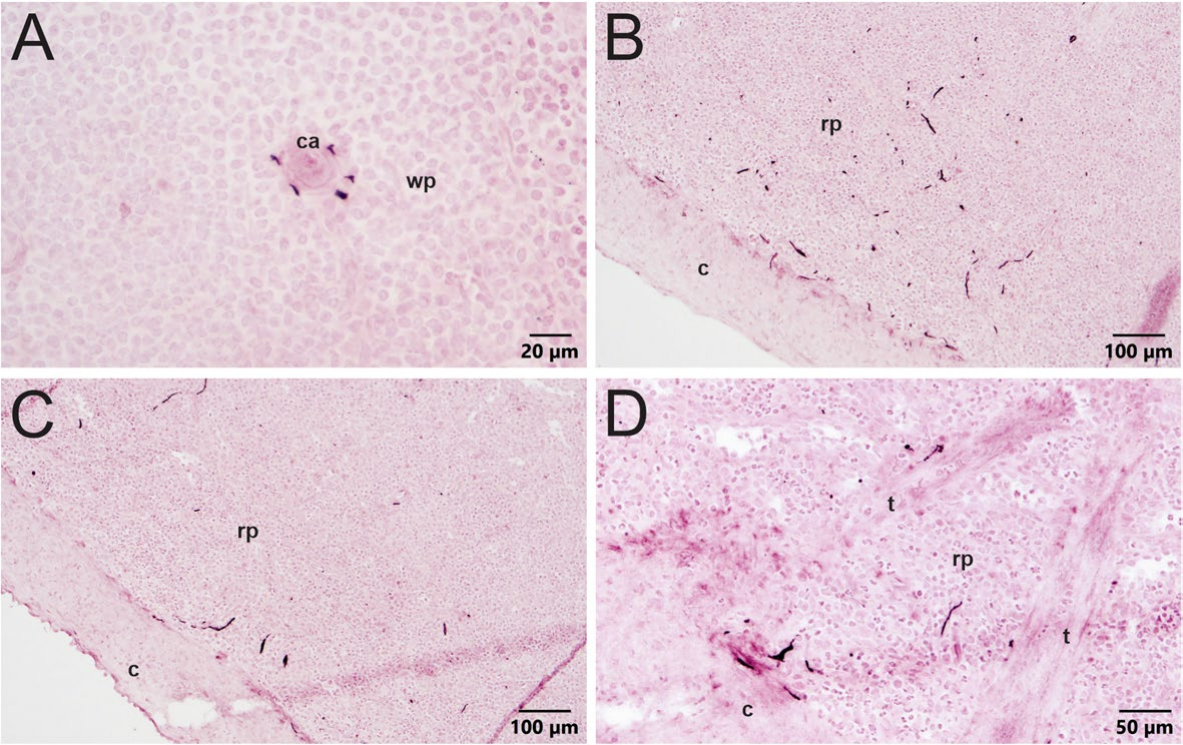
TH staining of spleen section from donor 002 (VIP chromogen and citrate buffer antigen retrieval). **A** Sympathetic innervation of a central arteriole (ca.) cut in cross section. TH + nerves extend further into the white pulp (wp) than observed in other donor spleens. **B** Sympathetic innervation of the red pulp (rp) near the capsule (c) in the hilum region. Note the capsule has no innervation. **C** Fewer TH + nerves occur in the red pulp of a sample from the pole region of the spleen. **D** A few sympathetic nerves run parallel to trabeculae (t) extending from the capsule into the red pulp

The spleen from donor 002 differed from the above pattern in that slightly more sympathetic nerves were detected overall, and more TH + nerve fibers penetrated a short distance into the white pulp (Fig. 4A). This spleen had a clear presence of TH + nerve fibers in red pulp regions, under the capsule, and adjacent to trabeculae (Fig. 4B-D).

### The abundance of noradrenergic nerves is substantially higher in human heart and porcine spleen compared to human spleen

**Fig. 5:**
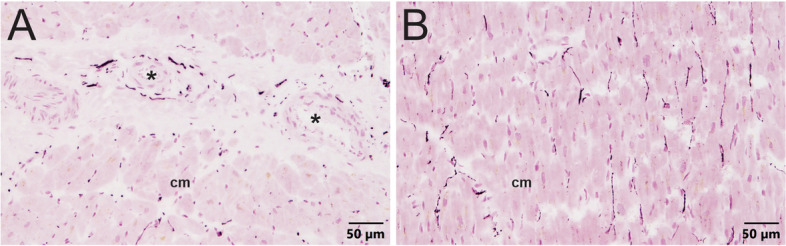
Localization of noradrenergic nerves in normal human left ventricle. **A** Sympathetic nerves occur in the adventitia of a coronary artery (*) and appear as puncta around cardiac myocytes (cm) cut in cross section. **B** TH + nerves run parallel to cardiac myocytes (cm) cut longitudinally

Given the limited distribution of noradrenergic nerves detected in human spleen sections, we used the same immunostaining methods to evaluate sympathetic innervation of two positive control tissues: human heart and porcine spleen. Sections of left ventricular muscle from normal human heart contained many noradrenergic nerve fibers that were closely associated with arteries and myocytes (Fig. 5). This finding contrasts with the results for human spleen and establishes the efficacy of our antibody and method for detecting noradrenergic nerves in human tissue.

**Fig. 6:**
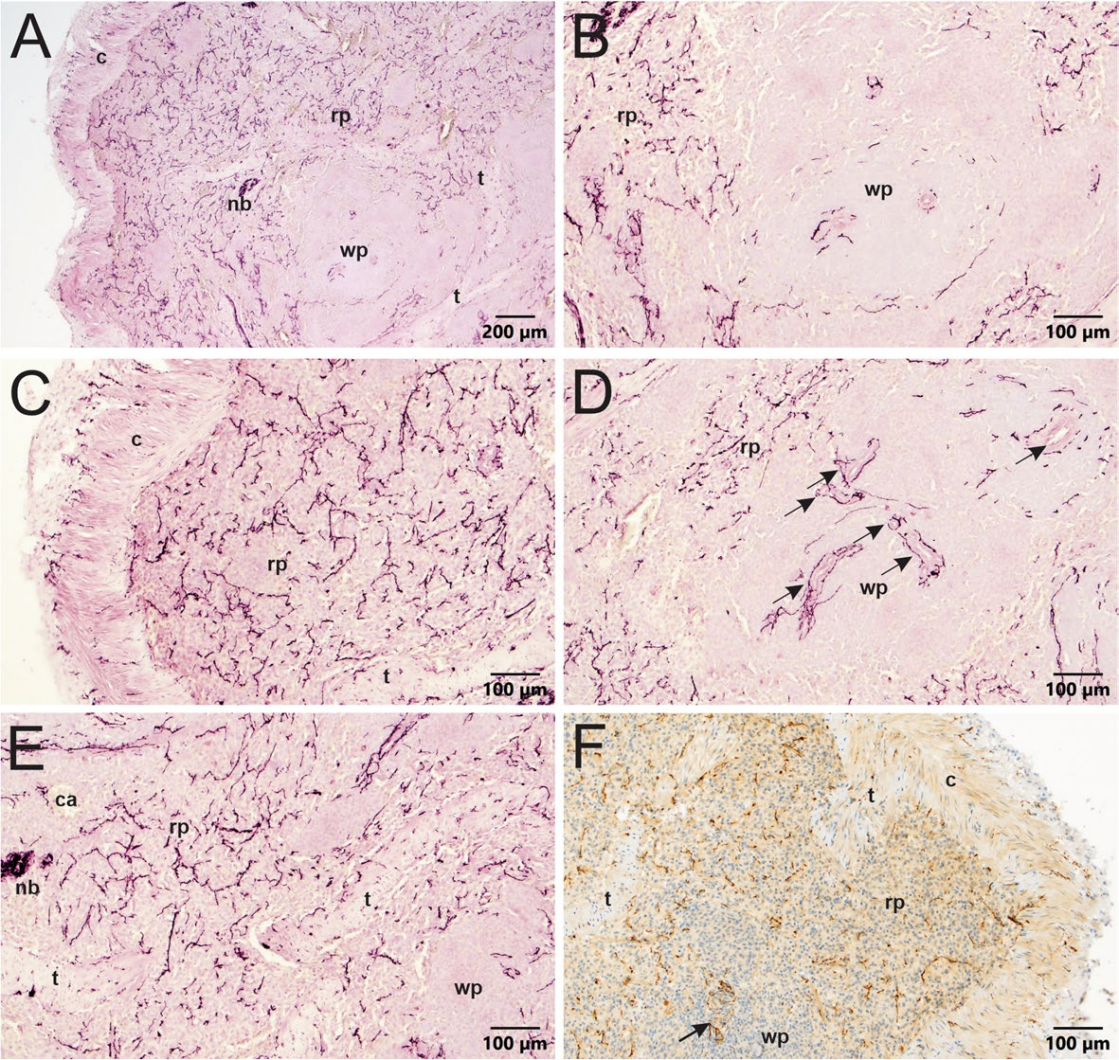
Regional distribution of abundant TH + nerves in porcine spleen. Sections shown in A-E were developed using VIP purple chromogen, and section in F was developed using DAB brown chromogen and counterstained with hematoxylin. **A** Low magnification image showing sympathetic innervation in capsule (c) and nearby parenchyma. TH + nerve fibers are most evident in the capsule and red pulp (rp), although some also occur in the white pulp (wp). Trabeculae (t) extend from the capsule into the red pulp. **B** Higher magnification of white pulp region of panel A showing TH + nerves around the central arterioles of the white pulp and in the red pulp. **C** Image showing TH + nerves in the capsule extending toward the heavily innervated red pulp. **D** Central arterioles (indicated with arrows) are surrounded by TH + nerves that run in the adventitia and extend into the white pulp. **E** Image illustrating the contrast of innervation in the white pulp, red pulp, and trabeculae. Red pulp is heavily innervated throughout. Nerves in the white pulp mainly extend from the central arteriole. TH + nerves occur within and along trabeculae. Nerve bundles (nb) are also common in the parenchyma of pig spleens. **F** Brown TH + nerves in parenchyma near the capsule (c) and trabeculae (t). Arrow indicates central arteriole surrounded by nerves. Note nerves in trabecula exiting the capsule

Noradrenergic innervation of porcine spleen was evaluated because the pig is a large animal species often used as a model for human biological systems and pathology (Dacey et al., 2022, Hanna et al., 2021, Sokal et al., 2021). We found that TH + nerve fibers were very dense in sections of porcine spleen and had a broad distribution that included arteries, white pulp, and red pulp (Fig. 6, Additional file 2). Noradrenergic innervation occurred in all arteries, and several nerve fibers penetrated well into the surrounding white pulp (Fig. 6A, B, and D). However, TH + nerves were far more abundant throughout the red pulp (Fig. 6). Both the capsule and trabeculae also received prominent innervation (Fig. 6). Myocardial infarction did not affect noradrenergic innervation of the spleen, at least at the 6 week survival time.

### The sympathetic co-transmitter NPY occurs in nerves and non-neuronal cells in human spleen

**Fig. 7:**
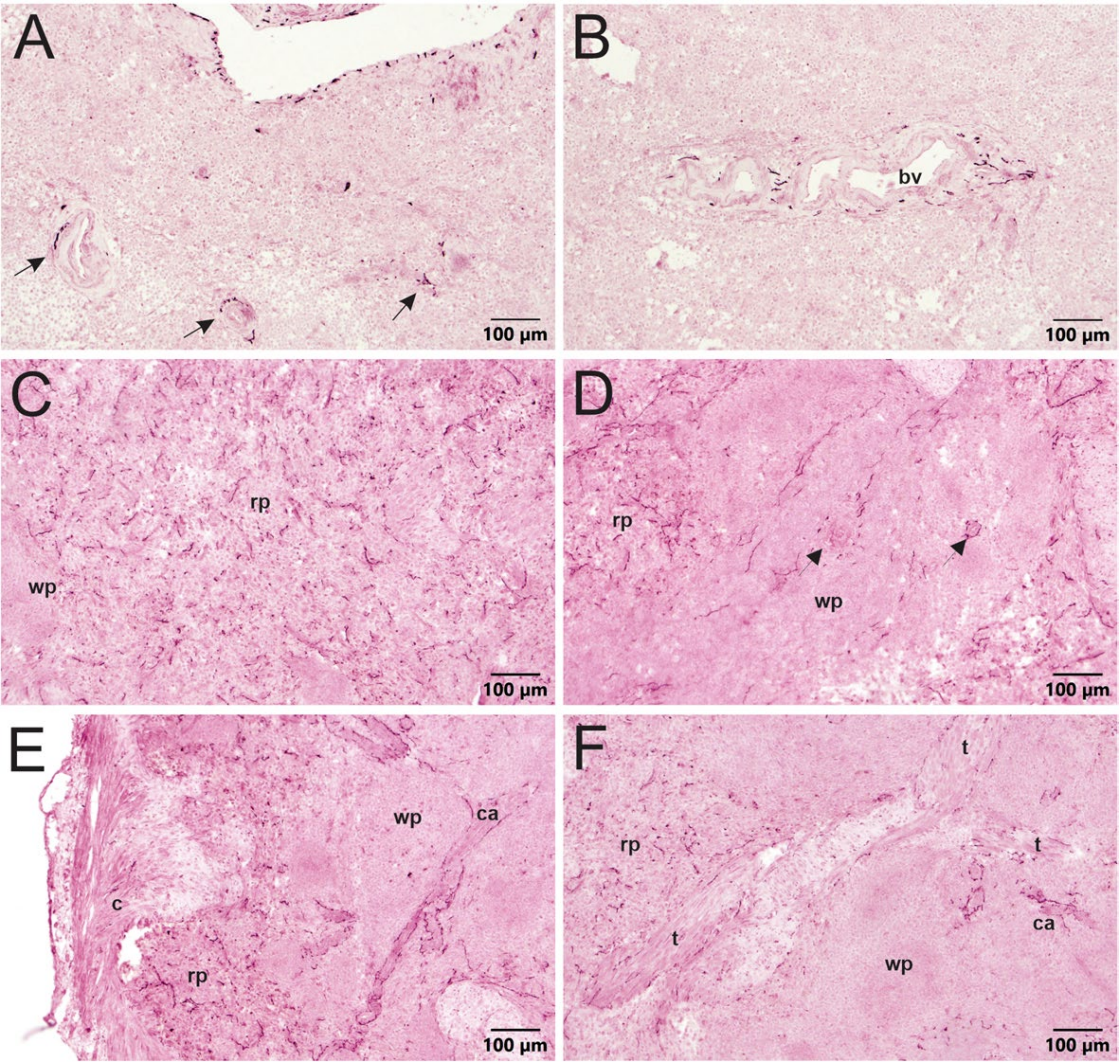
A comparison of NPY staining in sections of human (**A**-**B**) and porcine (**C**-**F**) spleen. NPY + sympathetic nerves are associated with splenic vasculature, like TH, in human spleen. **A** NPY + nerve staining around central arterioles (arrows) in section from donor 002 spleen. Few nerves extend a short distance into the surrounding parenchyma. **B** NPY + nerves around a central arteriole in section from donor 004 spleen. **C** Robust staining for NPY + nerves throughout the red pulp (rp) of pig spleen. White pulp (wp) has considerably less NPY staining. **D** Sympathetic nerves stain for NPY near and extending from central arterioles, indicated by arrows, within the white pulp. **E** The capsule does not contain NPY + innervation, but the red pulp beneath the capsule and the central arteries (ca.) extending from the capsule are heavily innervated. **F** Sympathetic nerves run parallel to trabeculae (t) in pig spleen parenchyma

The presence of NPY, which is often co-localized with TH in noradrenergic nerves, was used to further investigate sympathetic innervation of the spleen (Lundberg et al., 1983, Lundberg et al., 1989). This neuropeptide was detected in perivascular nerves of all the human spleens as observed for TH staining (Fig. 7A and B), but fewer arteries contained such nerves. NPY + nerve fibers were much more abundant in porcine spleen and mirrored the distribution of TH + nerves (Fig. 7C-D, Additional file 3).

Some non-neuronal staining for NPY was also detected in spleens from three of the donors, and this was most apparent in human spleen 003. For this patient, NPY immunoreactivity occurred in littoral cells that line venous sinuses throughout the spleen (Additional file 4). Littoral cells are specialized endothelial cells with phagocytic function (Qiu et al., 2018).

### Human and pig spleens differ in the location and abundance of sites where noradrenergic nerves occur in proximity to T cells

**Fig. 8:**
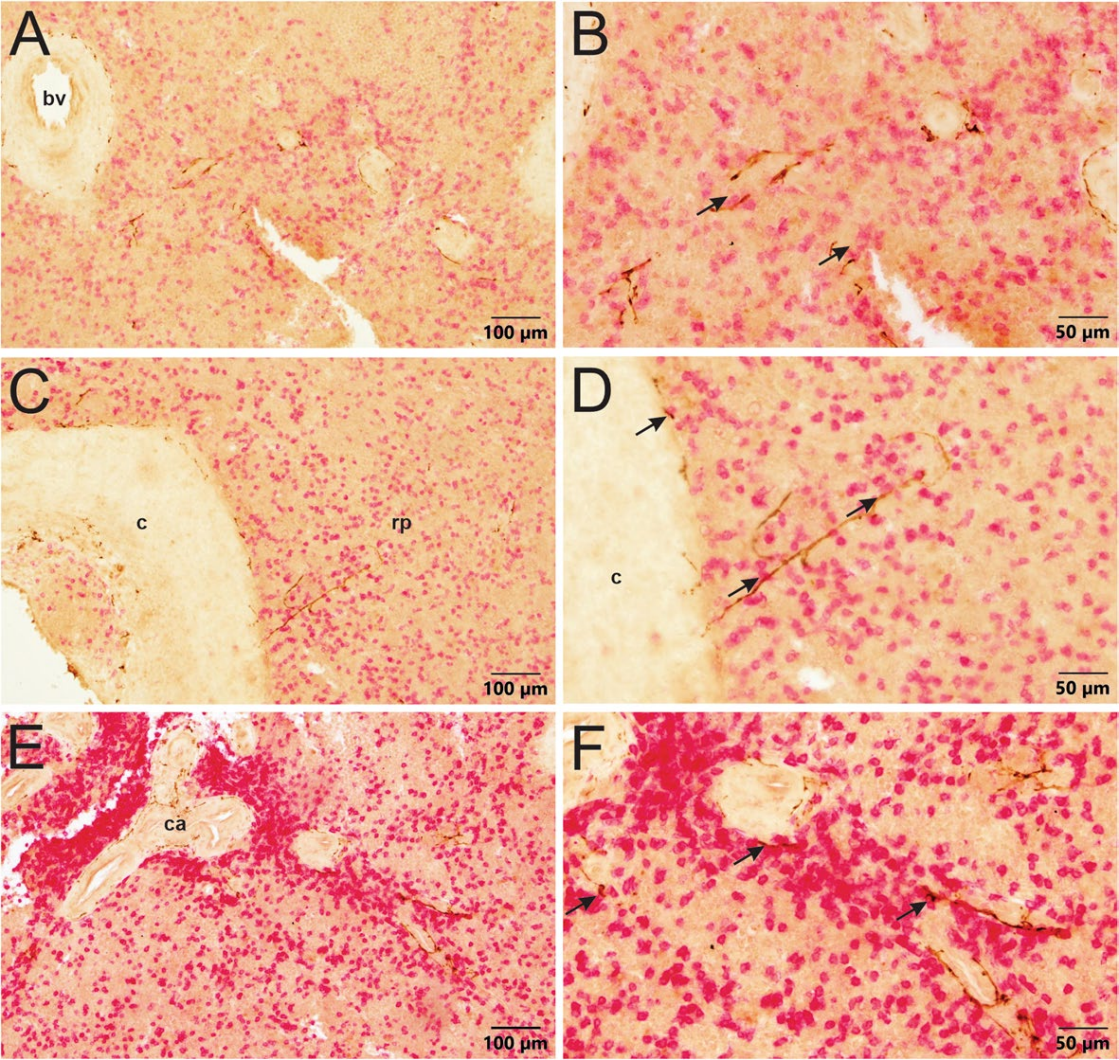
Double labeling for TH (brown) and CD3 (red) in sections from donor 002 spleen. **A** TH + nerves around blood vessels and throughout red pulp. CD3 + T leukocytes are scattered throughout the red pulp at a moderate rate. **B** Higher magnification image of panel A showing the close proximity of CD3 + T leukocytes with TH + nerves in red pulp, indicated by arrows. **C** TH + nerves extend from the capsule (c). **D** Higher magnification image of panel C showing the close association of TH + nerves (arrows) with CD3 + T leukocytes in this subcapsular region. **E** CD3 + T leukocytes are densely packed around a central arteriole (ca.) cut longitudinally. TH + nerves hug the blood vessels in the parenchyma. **F** Higher magnification image of panel E showing the close association of CD3 + T leukocytes and TH + nerves around blood vessels in the PALS

**Fig. 9:**
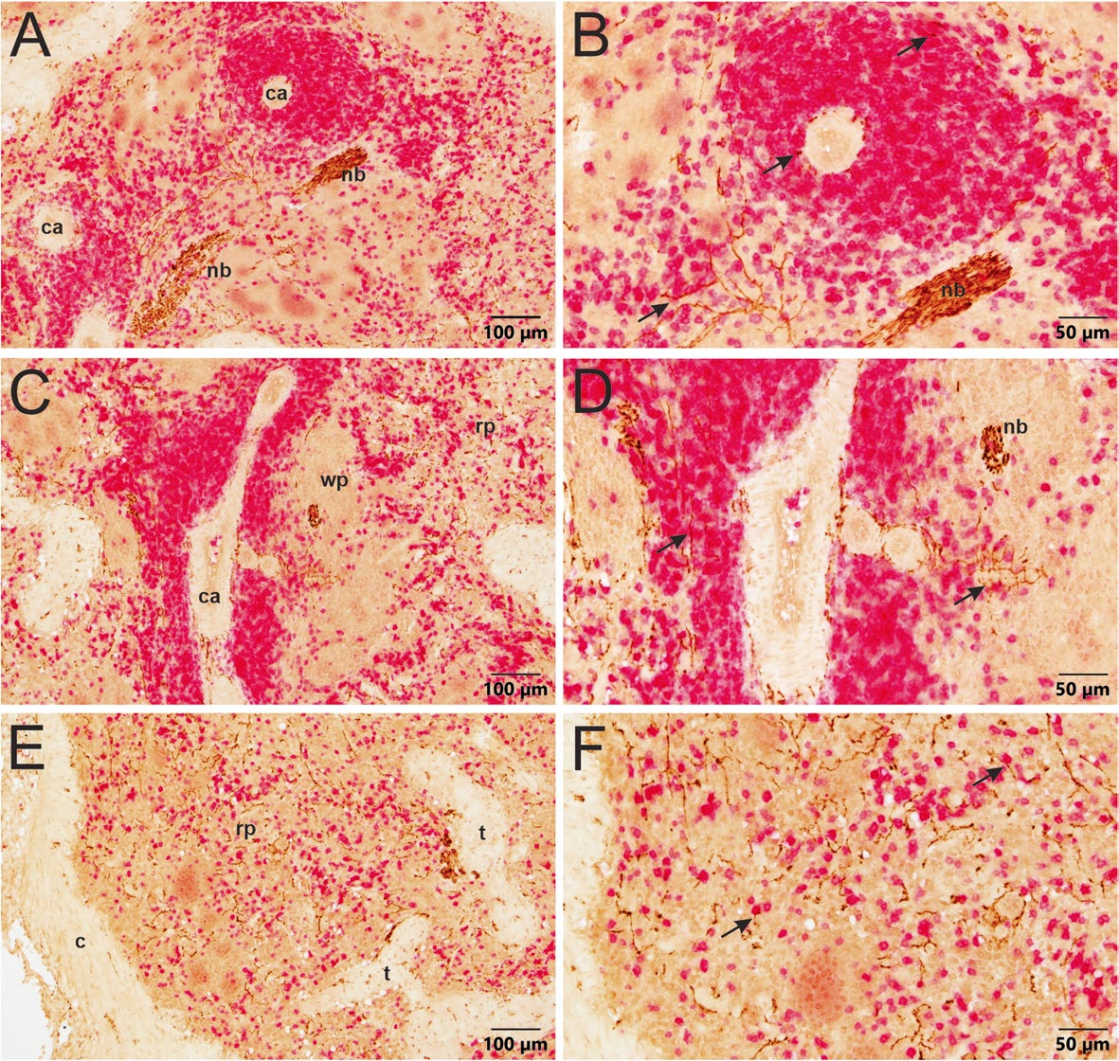
Double-labeling IHC in porcine spleen tissue for TH (brown) and CD3 (red). **A** CD3 + T leukocytes are densely packed around central arterioles (ca.). TH + nerves extend from the nerve bundles (nb) into the surrounding parenchyma. **B** Higher magnification image of panel A showing the close association of TH + nerves and CD3 + T leukocytes both around the central arteriole and in the surrounding parenchyma, indicated by arrows. **C** TH + nerves and CD3 + T leukocytes are tightly packed around the central artery (ca.) and loosely packed throughout the red pulp (rp). **D** Higher magnification image of panel C showing the close association of TH + nerves with CD3 + T leukocytes around the central artery, indicated by arrows. Note the nerve bundle in the top right corner. **E** The capsule, and trabeculae of pig spleens have less innervation. However, the red pulp is rich with sympathetic innervation and leukocytes. **F** Higher magnification image of panel E, showing the close apposition of TH + nerves and CD3 + T cells in the red pulp beneath the capsule in pig spleens, indicated by arrows

To illustrate the relationship of sympathetic innervation with leukocytes in the spleen, human and porcine tissue sections were double-immunolabeled for TH and the pan T cell marker, CD3. For human spleens, TH + nerves at the outer edge of central arteries often traveled very close to CD3 + cells located immediately adjacent to the blood vessel (Fig. 8). Extension of nerve fibers into the white pulp was not observed in double labeling experiments. However, some TH + nerve fibers were detected in subcapsular red pulp of donor 002, and these fibers traveled near CD3 + cells and other leukocytes (Fig. 8). In marked contrast, TH + nerve fibers were closely associated with T cells and other leukocytes in the white and red pulp of porcine spleen (Fig. 9).

## Discussion

The primary goal of this study was to illustrate the full extent of noradrenergic innervation of the human spleen and its relationship with splenic leukocytes, particularly T cells. This is important because the current model of the CAP, which was developed largely in rodent models, envisions a close association of sympathetic nerves with cholinergic T cells in the white pulp. Previous research performed with human tissue found that noradrenergic innervation of human spleen is mainly localized to the arterial vasculature and very sparse in the white pulp compared to that of rodent spleens (Cleypool et al., 2021, Hoover et al., 2017, Verlinden et al., 2019). Our findings support this view of sparse innervation of splenic parenchyma by using donor tissue immediately fixed while still viable and treated with two different antigen retrieval protocols. Specifically, TH + nerves occurred almost exclusively in the adventitia of arterial vessels and only rarely made short excursions into surrounding white pulp. However, the outermost perivascular nerves often occurred close to leukocytes at the immediate border of blood vessels. Some of the latter were CD3 + leukocytes. While this is the most common pattern, the spleen from one donor had a slightly broader distribution of TH + nerves, suggesting some degree of variation can occur between individuals. Sparse noradrenergic innervation of the spleen contrasts with abundant innervation of human left ventricular samples processed in the same way. Additional innervation was not revealed in sections stained for the co-transmitter, NPY. Using the same methodology, we found that porcine spleen had extremely dense noradrenergic/NPY innervation, particularly throughout the red pulp, which again differs from the pattern for rodents, which have sparse innervation of red pulp (Bellinger et al., 1989, Felten et al., 1985, Murray et al., 2017). Thus, there are major species-specific variations in sympathetic innervation of the spleen, with rodents, pigs, and humans having distinct patterns or regional innervation.

Several previous immunohistochemical studies evaluated sympathetic innervation of human spleen, using primarily archived autopsy samples and a limited number of fresh frozen samples (Cleypool et al., 2021, Hoover et al., 2017, Verlinden et al., 2019). This work was consistent in finding that a vast majority of noradrenergic nerves in the human spleen occurred around the arterial vasculature, where they were localized to perivascular adventitia. Two studies found a variable number of short nerve projections into the periarteriolar lymphatic sheath (PALS) where they occurred near leukocytes (Cleypool et al., 2021, Hoover et al., 2017). Cleypool et al. established that some of these leukocytes were CD3 + T cells and that well over half of the PALSs evaluated contained some noradrenergic nerves. However, such innervation of PALS and occasional nerves found at other sites in the parenchyma and capsule are quantitatively minor compared to the abundance of perivascular nerves. This conclusion is supported by our results as donor tissue was obtained with rapid preservation, confirming that these findings represent true innervation patterns rather than limitations of autopsy studies or delayed fixation. The limited innervation of human spleen stands in marked contrast to the abundant innervation we found in porcine spleens fixed and stained using the same protocol. We further evaluated human and porcine spleens for the sympathetic co-transmitter, NPY. To our knowledge this is the first demonstration of NPY + nerves in human spleens, and it was localized to perivascular nerves. In contrast, NPY + nerves in porcine spleen had the same extensive distribution as TH + nerves. These findings for porcine spleen concur with earlier reports, which established very dense noradrenergic innervation of porcine spleen and the presence of NPY in these nerves (Lundberg et al., 1988, Lundberg et al., 1989). These finding are likely to have functional implications for recent work that showed a robust CAP response to both cervical vagal and splenic nerve stimulation in a porcine model (Donegà et al. 2021, Sokal et al., 2021).

Vasoconstrictor and immunomodulatory effects have been described for NPY, but the latter have not been studied for intact spleen (Bedoui et al., 2003, Elenkov et al., 2000, Lundberg et al., 1988, Lundberg et al., 1989). While the neuronal localization of NPY was limited to perivascular nerves in human spleens, variable localization to non-neuronal cells also occurred, especially in donor 003 where extensive labeling of reticular endothelial cells was observed. This may be related to the patient’s diagnosis of arthritis, since increased levels of NPY have been detected in the serum and at sites of inflammation in this chronic inflammatory disease (Bedoui et al., 2003, Kopec-Medrek et al., 2012, Wang et al., 2014). Further work is needed to determine if these cells produce or sequester NPY.

Innervation of the spleen has been a topic of interest for many decades, beginning in earnest with early studies of neurochemical transmission and the development of fluorescence histochemical methods for identifying catecholamines in tissue. This interest expanded with the availability of immunohistochemical methods and the discovery of direct sympathetic effects on the immune system. Accordingly, there is now a wealth of data on the presence and distribution of sympathetic nerves in the spleen for a wide range of animal species. Three basic patterns appear from this data. Based on the current study and previous work, humans appear at the low end of the spectrum, with abundant innervation of the arterial vasculature and sparse innervation of the PALS, red pulp, and capsule. Innervation of cat spleen has a pattern like that of human spleen, but this species has more nerves associated with the capsule (Fillenz, 1970, Lundberg et al., 1985). Small species, such as mouse, rat, and guinea pig, have the same basic pattern but also have sympathetic nerves distributed throughout the PALS (Bellinger et al., 1989, Felten et al., 1985, Murray et al., 2017). A network of noradrenergic nerve fibers in the capsule and trabeculae has also been described for rats and guinea pigs (Jobling, 1994). The upper end of the spectrum is represented by canine and porcine spleens, which have extremely dense and widespread sympathetic innervations that includes the arterial vasculature, capsule, trabeculae, and the parenchyma (Dahlström and Zetterström, 1965, Dahlström et al., 1965, Lundberg et al., 1988). The distribution of nerves within the parenchyma is particularly interesting in these species, since nerve density in the red pulp is overwhelming compared to that in the white pulp. Nevertheless, the innervation pattern of porcine white pulp observed in the present study is like that in rodent white pulp. Thus, the distribution and abundance of sympathetic nerves varies substantially between species and is not related to body mass. This point is emphasized by studies showing that innervation of the Beluga whale spleen most closely matches that seen in small animals (Romano et al., 1994).

Sympathetic nerves can have multiple effector functions within the spleen and some of these depend on the pattern of innervation. This is clearly the case for regulation of spleen hemodynamics. Dense noradrenergic innervation of the arterial vasculature is common to all species studied and regulates perfusion via arterial constriction. Sympathetic control of this system has been studied extensively in experiments using isolated cat and dog spleens (Ayers et al., 1972, Blakeley, 1968, Cripps and Dearnaley, 1972, Hertting and Suko, 1966, Thoenen et al., 1964) and in early work with isolated human spleens (Ayers et al., 1972). Stimulation of the splenic nerve in these preparations causes a frequency-dependent increase in perfusion pressure that is mediated by release of NE. Studies using porcine spleens showed that higher frequency stimulation also causes release of NPY, which contributes to vasoconstriction (Lundberg et al., 1986, Lundberg et al., 1989). Our observation of perivascular NPY nerves in human spleens suggests a similar function to that in the pig. Many of these studies also evaluated stimulation-evoked contraction of the splenic capsule, which manifested as a decrease in spleen volume. Such changes are indicative of increased venous flow, and occurred in cat, dog, and porcine spleens but not in the human (Ayers et al., 1972, Blakeley, 1968, Lundberg et al., 1985, Sokal et al., 2021). The lack of response in humans can be explained by the near absence of noradrenergic nerves in the capsule, as shown here and in previous work (Cleypool et al., 2021, Hoover et al., 2017, Verlinden et al., 2019). How or if between-species variation in sympathetic innervation of the spleen impacts its modulation of immune function still needs to be clarified, especially in the context of the CAP.

Anatomical, cellular, and biochemical aspects of the CAP in the spleen have been elucidated entirely using mouse and rat models and focus on sympathetic innervation of the white pulp. Close juxtaposition of noradrenergic nerves with cholinergic T cells was proposed initially (Felten and Olschowka, 1987, Rosas-Ballina et al., 2011), but subsequent work showed that this is rare in mouse spleen (Murray et al., 2017). So, diffusion of NE to its target T cells appears to be required even in the mouse. Likewise, diffusion of ACh is required to reach its target population of macrophages. Our findings and previous work on innervation of porcine spleen suggest a variation on the theme, since innervation of the red pulp dominates in this species (Lundberg et al., 1988). Importantly, our work shows that this region contains many T cells, and it is also known to be rich in macrophages (den Haan and Kraal, 2012, Nagelkerke et al., 2018). However, significant diffusion of NE must be required for the CAP to work in species like the human and cat, which have sparse innervation of both white and red pulp.

Cleypool and colleagues provided an excellent analysis of potential mechanisms for NE dynamics in the human spleen that would support cholinergic anti-inflammatory mechanisms as well as other neuromodulatory effects (Cleypool et al., 2021). If such diffusion-dependent mechanisms exist, then NE should be present in the venous compartment of the spleen. In fact, previous studies have detected NE in the venous effluent of isolated dog, pig, cat, and human spleen after splenic nerve stimulation (Ayers et al., 1972, Cripps and Dearnaley, 1972, Farmer, 1966, Hertting and Suko, 1966, Lundberg et al., 1986, Schoups et al., 1988, Sokal et al., 2021, Thoenen et al., 1964). This is not surprising for the pig and dog, given their abundant innervation of red pulp. However, the dominant perivascular localization of sympathetic nerves in cat and human spleens suggests that NE must travel a substantial distance to occur in the venous effluent.

While there were a limited number of donor spleens available for this study, they were sufficient to achieve our immediate experimental goals. Importantly, our sample population included females and males, with an overall age range of about 30 years. Furthermore, many of these donors had comorbidities that could be targeted with neuromodulation therapy. Finally, these samples were collected and fixed immediately after patient demise, increasing the power and accuracy of localization studies in these human samples.

Several recent clinical trials have applied VNS with some success for treatment of rheumatoid arthritis, inflammatory bowel disease, and Crohn’s disease (Pavlov and Tracey, 2022). Based on preclinical studies of model systems, it seems likely that activation of the CAP contributes to these positive clinical results (Hoover, 2017, Pavlov and Tracey, 2022). Given the dominant perivascular localization of noradrenergic nerves in human spleen, identified in this and prior studies (Cleypool et al., 2021, Hoover et al., 2017, Verlinden et al., 2019), greater engagement of sympathetic nerves and the CAP might be achieved by direct stimulation of the splenic nerve compared to cervical VNS. Recent studies of porcine and human models support the feasibility of such an approach with sophisticated cuff electrodes (Donegà et al. 2021, Gupta et al., 2020, Sokal et al., 2021). Further innovative preclinical studies indicate that focused ultrasound stimulation applied to the spleen is an effective way to activate the CAP non-invasively (Cotero et al., 2019a, Cotero et al., 2019b, Zachs et al., 2019). For application of this approach to humans, our results suggest targeting the hilum region where sympathetic nerve bundles enter the spleen would be optimum.

## Conclusion

In conclusion, results from our experiments support the view that sympathetic innervation of the human spleen is localized primarily to the adventitia of arterial vessels, even using donor tissue that was fixed immediately after collection. This finding agrees with previous studies of human spleen innervation and correlates with early data that demonstrated sympathetic nerve stimulation constricts splenic arteries but does not affect venous outflow from the human spleen. We also show a similar but less abundant distribution of NPY, a well-known sympathetic co-transmitter with vasoconstrictor properties. The restricted distribution of noradrenergic nerves in human spleens contrasts with abundant innervation of porcine spleen found in the same experiments using identical methods. This work and review of the literature show that the pattern of sympathetic innervation can vary markedly between species. Diffusion of NE and ACh within the spleen likely plays an important role in neuromodulation of immune function in all species but would be especially important for humans. Our findings for localization of sympathetic nerves in the human spleen have important implications on focused targeting of the CAP for treatment of chronic inflammatory diseases.

## Supplementary Information

**Additional file 1.** Montage image showing a large section of human spleen tissue stained for TH. TH + nerve fibers occurred mainly around the arterial vasculature and rarely in the white pulp (wp) or red pulp (rp). Sympathetic nerve bundles (nb) were occasionally seen in large arteries. Arrows indicate central arterioles. Montage was created by stitching multiple 10X images using an Olympus BX41 microscope equipped with an Olympus DP74 digital camera and cellSens Dimension software.

**Additional file 2. **Montage image showing a large section of porcine spleen stained for TH. TH + nerve fibers were more prevalent in the red pulp (rp) than the white pulp (wp), trabeculae (t), or capsule (c). Sympathetic nerve bundles (nb) are scattered throughout the section. Montage was created by stitching multiple 10X images using an Olympus BX41 microscope equipped with an Olympus DP74 digital camera and cellSens Dimension software.

**Additional file 3.** Montage image showing a large section of porcine spleen stained for NPY. NPY + nerve fibers were found in all regions of pig spleen, with red pulp being the most densely innervated. White pulp (wp), red pulp (rp), and trabeculae (t) are labeled. Montage was created by stitching multiple 10X images using an Olympus BX41 microscope equipped with an Olympus DP74 digital camera and cellSens Dimension software.

**Additional file 4. **Spleen from donor 003 exhibited a unique, robust pattern of NPY staining. (A) Image showing NPY nerve staining around a splenic artery (sa) and staining within surrounding red pulp (rp). Arrow indicates NPY staining of littoral cells. (B) Image showing NPY staining of littoral cells (arrow) in the subcapsular region. Note that the trabeculae (t) and capsule (c) are largely free from NPY staining while the red pulp shows dense staining. (C) Image showing lack of innervation within the white pulp (wp) and limited innervation around a central arteriole (ca.). (D) Image showing intense and abundant staining of littoral cells (arrows) within the red pulp.

## Data Availability

The datasets used and/or analyzed during the current study are available from the corresponding author on reasonable request.

## Abbreviations

BM: Bone marrow
CAP: Cholinergic anti-inflammatory pathway
NE: norepinephrine
ACh: Acetylcholine
TH: Tyrosine hydroxylase
IHC: Immunohistochemistry
NPY: Neuropeptide Y
EDTA: Ethylenediaminetetraacetic acid
ABC: Avidin-Biotin Complex
HRP: Horseradish peroxidase
BSA: Bovine serum albumin
DAB: Diaminobenzidine
H&E: Hematoxylin and eosin
PALS: Periarteriolar lymphatic sheath
wp: White pulp
ca: Central artery/arteriole
sa: Splenic artery
rp: Red pulp
c: Capsule
cm: Cardiac myocytes
t: Trabeculae
bv: Blood vessel
nb: Nerve bundle
VNS: Vagal nerve stimulation

## References

[CR1] Ayers AB, Davies BN, Withrington PG (1972). Responses of the isolated, perfused human spleen to sympathetic nerve stimulation, catecholamines and polypeptides. Br J Pharmacol.

[CR2] Bedoui S, Kawamura N, Straub RH, Pabst R, Yamamura T, von Hörsten S (2003). Relevance of neuropeptide Y for the neuroimmune crosstalk. J Neuroimmunol.

[CR3] Bellinger DL, Lorton D (2014). Autonomic regulation of cellular immune function. Auton Neurosci.

[CR4] Bellinger DL, Felten SY, Lorton D, Felten DL (1989). Origin of noradrenergic innervation of the spleen in rats. Brain Behav Immun.

[CR5] Blakeley AG (1968). The responses of the spleen to nerve stimulation in relation to the frequency of splenic nerve discharge. Proc R Soc Lond B Biol Sci.

[CR6] Cleypool CGJ, Brinkman DJ, Mackaaij C (2021). Age-related variation in sympathetic nerve distribution in the human spleen. Front Neurosci.

[CR7] Cotero V, Fan Y, Tsaava T (2019). Noninvasive sub-organ ultrasound stimulation for targeted neuromodulation. Nat Commun.

[CR8] Cotero V, Graf J, Zachs DP (2019). Peripheral focused Ultrasound Stimulation (pFUS): New Competitor in Pharmaceutical Markets. SLAS Technol.

[CR9] Cripps H, Dearnaley DP (1972). Vascular responses and noradrenaline overflows in the isolated blood-perfused cat spleen: some effects of cocaine, normetanephrine and -blocking agents. J Physiol.

[CR10] Dacey M, Salahudeen O, Swid MA, Carlson C, Shivkumar K, Ardell JL (2022). Structural and function organization of intrathoracic extracardiac autonomic projections to the porcine heart: implications for targeted neuromodulation therapy. Heart Rhythm.

[CR11] Dahlström AB, Zetterström BE (1965). Noradrenalin stores in nerve terminals of the spleen: changes during hemorrhagic shock. Science.

[CR12] Dahlström A, Mya-Tu M, Fuxe K, Zetterström BE (1965). Observations on adrenergic innervation of dog heart. Am J Physiol.

[CR13] den Haan JM, Kraal G (2012). Innate immune functions of macrophage subpopulations in the spleen. J Innate Immun.

[CR14] Donegà M, Fjordbakk CT, Kirk J (2021). Human-relevant near-organ neuromodulation of the immune system via the splenic nerve. Proc Natl Acad Sci U S A.

[CR15] Elenkov IJ, Wilder RL, Chrousos GP, Vizi ES (2000). The sympathetic nerve–an integrative interface between two supersystems: the brain and the immune system. Pharmacol Rev.

[CR16] Falvey A, Metz CN, Tracey KJ, Pavlov VA (2022). Peripheral nerve stimulation and immunity: the expanding opportunities for providing mechanistic insight and therapeutic intervention. Int Immunol.

[CR17] Farmer JB (1966). Liberation of noradrenaline from the dog spleen. J Pharm Pharmacol.

[CR18] Felten SY, Olschowka J (1987). Noradrenergic sympathetic innervation of the spleen: II. Tyrosine hydroxylase (TH)-positive nerve terminals form synapticlike contacts on lymphocytes in the splenic white pulp. J Neurosci Res.

[CR19] Felten DL, Felten SY, Carlson SL, Olschowka JA, Livnat S (1985). Noradrenergic and peptidergic innervation of lymphoid tissue. J Immunol.

[CR20] Fillenz M (1970). The innervation of the cat spleen. Proc R Soc Lond B Biol Sci.

[CR21] Gupta I, Cassará AM, Tarotin I (2020). Quantification of clinically applicable stimulation parameters for precision near-organ neuromodulation of human splenic nerves. Commun Biol.

[CR22] Hanna P, Dacey MJ, Brennan J (2021). Innervation and neuronal control of the mammalian Sinoatrial Node a Comprehensive Atlas. Circ Res.

[CR23] Hertting G, Suko J (1966). Influence of angiotensin, vasopressin or changes in flow rate on vasoconstriction, changes in volume and [3H]-noradrenaline release following postganglionic sympathetic nerve stimulation in the isolated cat spleen. Br J Pharmacol Chemother.

[CR24] Hoover DB (2017). Cholinergic modulation of the immune system presents new approaches for treating inflammation. Pharmacol Ther.

[CR25] Hoover DB, Brown TC, Miller MK, Schweitzer JB, Williams DL (2017). Loss of sympathetic nerves in Spleens from Patients with End Stage Sepsis. Front Immunol.

[CR26] Jobling P (1994). Electrophysiological events during neuroeffector transmission in the spleen of guinea-pigs and rats. J Physiol.

[CR27] Jung WC, Levesque JP, Ruitenberg MJ (2017). It takes nerve to fight back: the significance of neural innervation of the bone marrow and spleen for immune function. Semin Cell Dev Biol.

[CR28] Kelly MJ, Breathnach C, Tracey KJ, Donnelly SC (2022). Manipulation of the inflammatory reflex as a therapeutic strategy. Cell Rep Med.

[CR29] Kopec-Medrek M, Kotulska A, Widuchowska M, Adamczak M, Więcek A, Kucharz EJ (2012). Plasma leptin and neuropeptide Y concentrations in patients with rheumatoid arthritis treated with infliximab, a TNF-α antagonist. Rheumatol Int.

[CR30] Krenacs L, Krenacs T, Stelkovics E, Raffeld M (2010). Heat-induced antigen retrieval for immunohistochemical reactions in routinely processed paraffin sections. Methods Mol Biol.

[CR31] Kressel AM, Tsaava T, Levine YA (2020). Identification of a brainstem locus that inhibits tumor necrosis factor. Proc Natl Acad Sci U S A.

[CR32] Lehner KR, Silverman HA, Addorisio ME (2019). Forebrain Cholinergic Signaling regulates Innate Immune responses and inflammation. Front Immunol.

[CR33] Lundberg JM, Terenius L, Hökfelt T, Goldstein M (1983). High levels of neuropeptide Y in peripheral noradrenergic neurons in various mammals including man. Neurosci Lett.

[CR34] Lundberg JM, Anggård A, Pernow J, Hökfelt T, Neuropeptide (1985). Y-, substance P- and VIP-immunoreactive nerves in cat spleen in relation to autonomic vascular and volume control. Cell Tissue Res.

[CR35] Lundberg JM, Rudehill A, Sollevi A, Theodorsson-Norheim E, Hamberger B (1986). Frequency- and reserpine-dependent chemical coding of sympathetic transmission: differential release of noradrenaline and neuropeptide Y from pig spleen. Neurosci Lett.

[CR36] Lundberg JM, Hemsén A, Rudehill A (1988). Neuropeptide Y- and alpha-adrenergic receptors in pig spleen: localization, binding characteristics, cyclic AMP effects and functional responses in control and denervated animals. Neuroscience.

[CR37] Lundberg JM, Rudehill A, Sollevi A, Hamberger B (1989). Evidence for co-transmitter role of neuropeptide Y in the pig spleen. Br J Pharmacol.

[CR38] Madden KS (2017). Sympathetic neural-immune interactions regulate hematopoiesis, thermoregulation and inflammation in mammals. Dev Comp Immunol.

[CR39] Murray K, Godinez DR, Brust-Mascher I, Miller EN, Gareau MG, Reardon C (2017). Neuroanatomy of the spleen: mapping the relationship between sympathetic neurons and lymphocytes. PLoS ONE.

[CR40] Murray K, Barboza M, Rude KM, Brust-Mascher I, Reardon C (2019). Functional circuitry of neuro-immune communication in the mesenteric lymph node and spleen. Brain Behav Immun.

[CR41] Murray K, Rude KM, Sladek J, Reardon C (2021). Divergence of neuroimmune circuits activated by afferent and efferent vagal nerve stimulation in the regulation of inflammation. J Physiol.

[CR42] Nagelkerke SQ, Bruggeman CW, den Haan JMM (2018). Red pulp macrophages in the human spleen are a distinct cell population with a unique expression of Fc-γ receptors. Blood Adv.

[CR43] Padro CJ, Sanders VM (2014). Neuroendocrine regulation of inflammation. Semin Immunol.

[CR44] Pavlov VA, Tracey KJ (2019). Bioelectronic medicine: updates, challenges and paths forward. [editorial]. Bioelectron Med.

[CR45] Pavlov VA, Tracey KJ (2022). Bioelectronic medicine: preclinical insights and clinical advances. Neuron.

[CR46] Qiu J, Salama ME, Hu CS, Li Y, Wang X, Hoffman R (2018). The characteristics of vessel lining cells in normal spleens and their role in the pathobiology of myelofibrosis. Blood Adv.

[CR47] Romano TA, Felten SY, Olschowka JA, Felten DL (1994). Noradrenergic and peptidergic innervation of lymphoid organs in the beluga, Delphinapterus leucas: an anatomical link between the nervous and immune systems. J Morphol.

[CR48] Rosas-Ballina M, Olofsson PS, Ochani M (2011). Acetylcholine-synthesizing T cells relay neural signals in a vagus nerve circuit. Science.

[CR49] Schoups AA, Saxena VK, Tombeur K, De Potter WP (1988). Facilitation of the release of noradrenaline and neuropeptide Y by the alpha 2-adrenoceptor blocking agents idazoxan and hydergine in the dog spleen. Life Sci.

[CR50] Sokal DM, McSloy A, Donegà M (2021). Splenic nerve Neuromodulation reduces inflammation and promotes resolution in chronically implanted Pigs. Front Immunol.

[CR51] Thoenen H, Huerlimann A, Haefely W (1964). The effect of sympathetic nerve stimulation on volume, vascular resistance, and norepinephrine output in the isolated perfused spleen of the cat, and its modification by cocaine. J Pharmacol Exp Ther.

[CR52] Verlinden TJM, van Dijk P, Hikspoors J, Herrler A, Lamers WH, Köhler SE (2019). Innervation of the human spleen: a complete hilum-embedding approach. Brain Behav Immun.

[CR53] Vida G, Peña G, Deitch EA, Ulloa L (2011). α7-cholinergic receptor mediates vagal induction of splenic norepinephrine. J Immunol.

[CR54] Vida G, Peña G, Kanashiro A (2011). β2-Adrenoreceptors of regulatory lymphocytes are essential for vagal neuromodulation of the innate immune system. FASEB J.

[CR55] Wang H, Yu M, Ochani M (2003). Nicotinic acetylcholine receptor alpha7 subunit is an essential regulator of inflammation. Nature.

[CR56] Wang L, Zhang L, Pan H, Peng S, Lv M, Lu WW (2014). Levels of neuropeptide Y in synovial fluid relate to pain in patients with knee osteoarthritis. BMC Musculoskelet Disord.

[CR57] Zachs DP, Offutt SJ, Graham RS (2019). Noninvasive ultrasound stimulation of the spleen to treat inflammatory arthritis. Nat Commun.

