# Supplementary figures and images for: Sympathetic innervation of human and porcine spleens: implications for between species variation in function
Source: Bioelectron Med. 2022 Dec 19;8:20. doi: 10.1186/s42234-022-00102-1 (PMC9762010; doi:10.1186/s42234-022-00102-1)

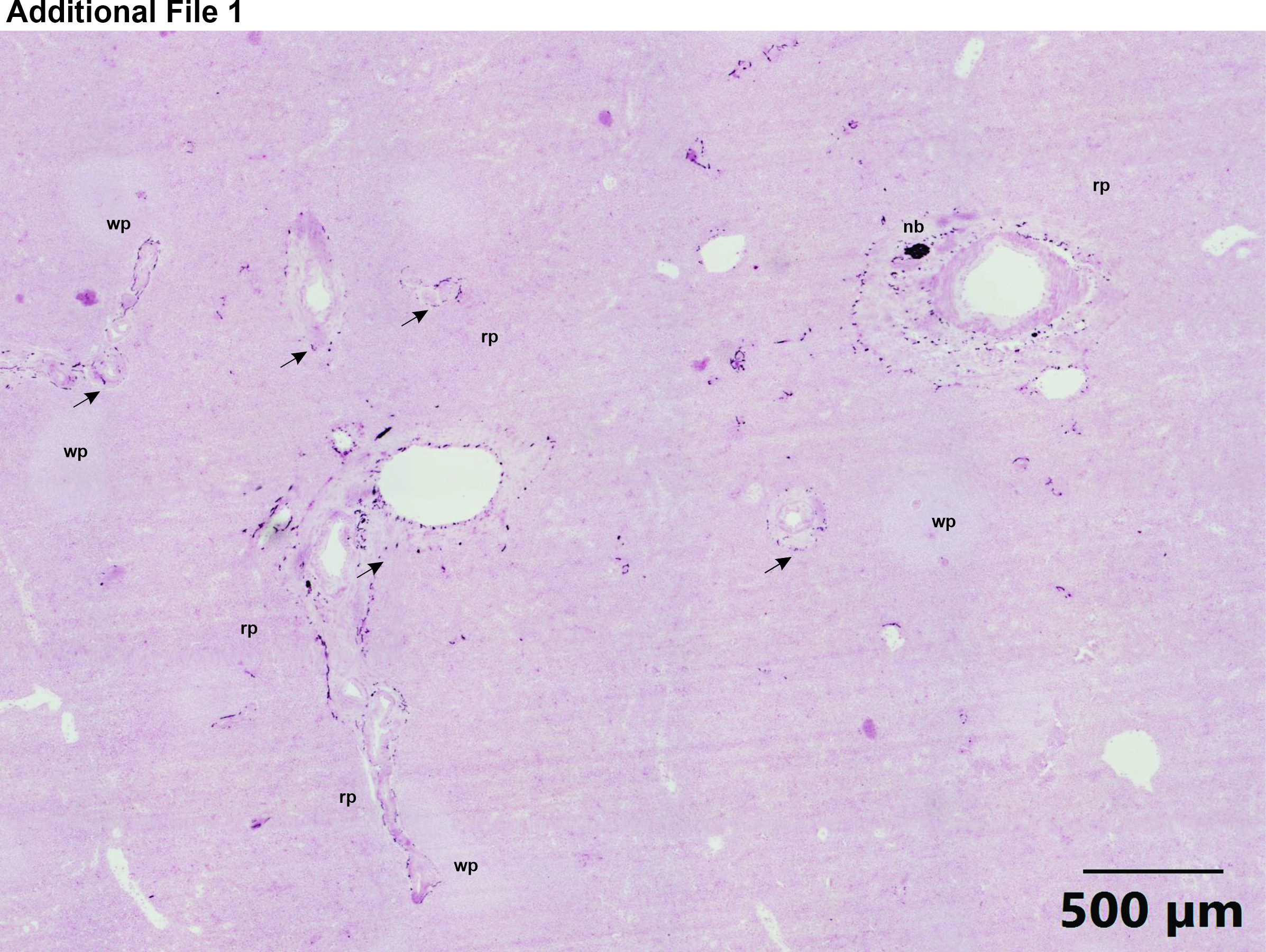

Supplement: Supplementary file 1 — Additional file 1. Montage image showing a large section of human spleen tissue stained for TH. TH + nerve fibers occurred mainly around the arterial vasculature and rarely in the white pulp (wp) or red pulp (rp). Sympathetic nerve bundles (nb) were occasionally seen in large arteries. Arrows indicate central arterioles. Montage was created by stitching multiple 10X images using an Olympus BX41 microscope equipped with an Olympus DP74 digital camera and cellSens Dimension software. [file 42234_2022_102_MOESM1_ESM.jpg]

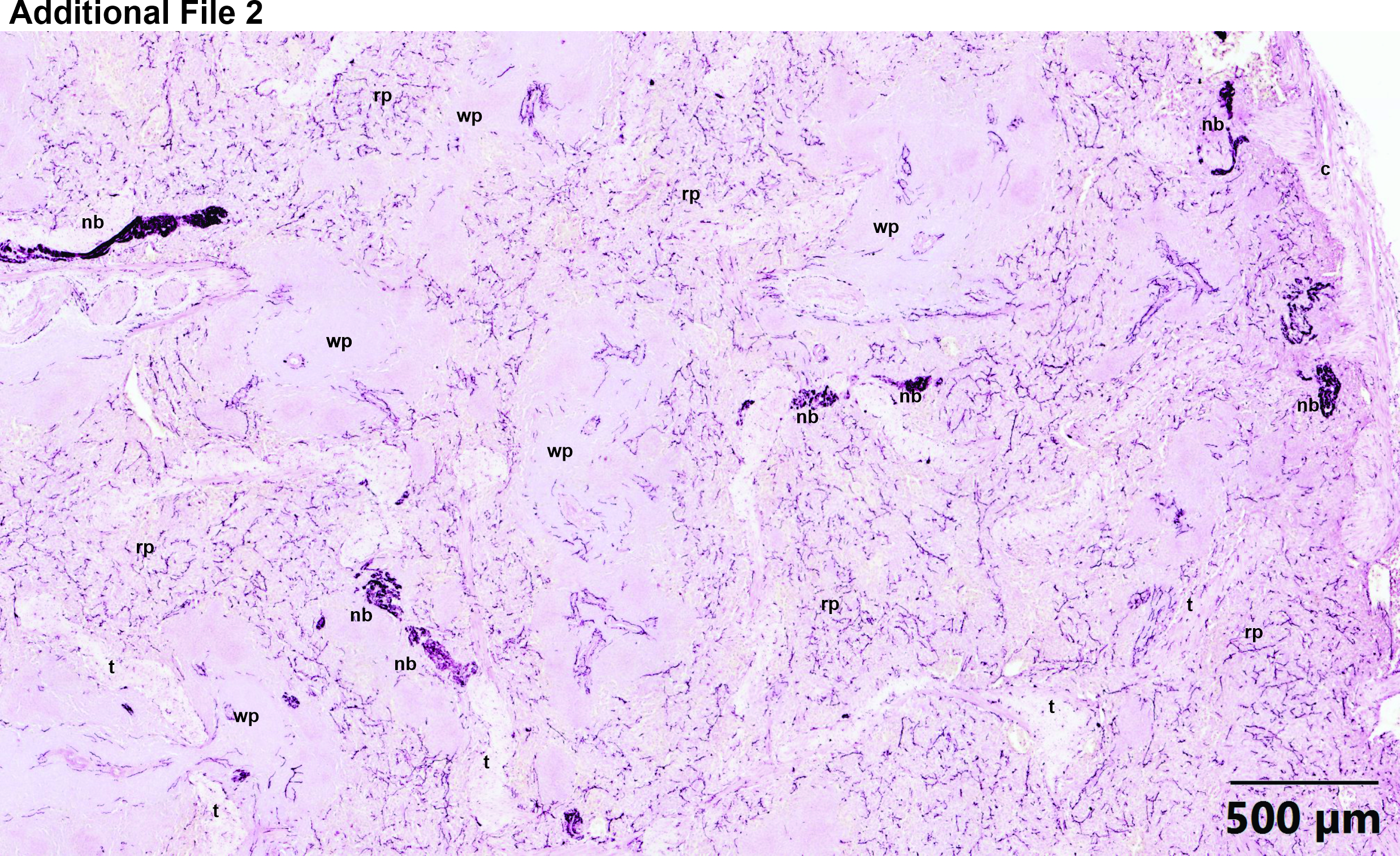

Supplement: Supplementary file 2 — Additional file 2. Montage image showing a large section of porcine spleen stained for TH. TH + nerve fibers were more prevalent in the red pulp (rp) than the white pulp (wp), trabeculae (t), or capsule (c). Sympathetic nerve bundles (nb) are scattered throughout the section. Montage was created by stitching multiple 10X images using an Olympus BX41 microscope equipped with an Olympus DP74 digital camera and cellSens Dimension software. [file 42234_2022_102_MOESM2_ESM.jpg]

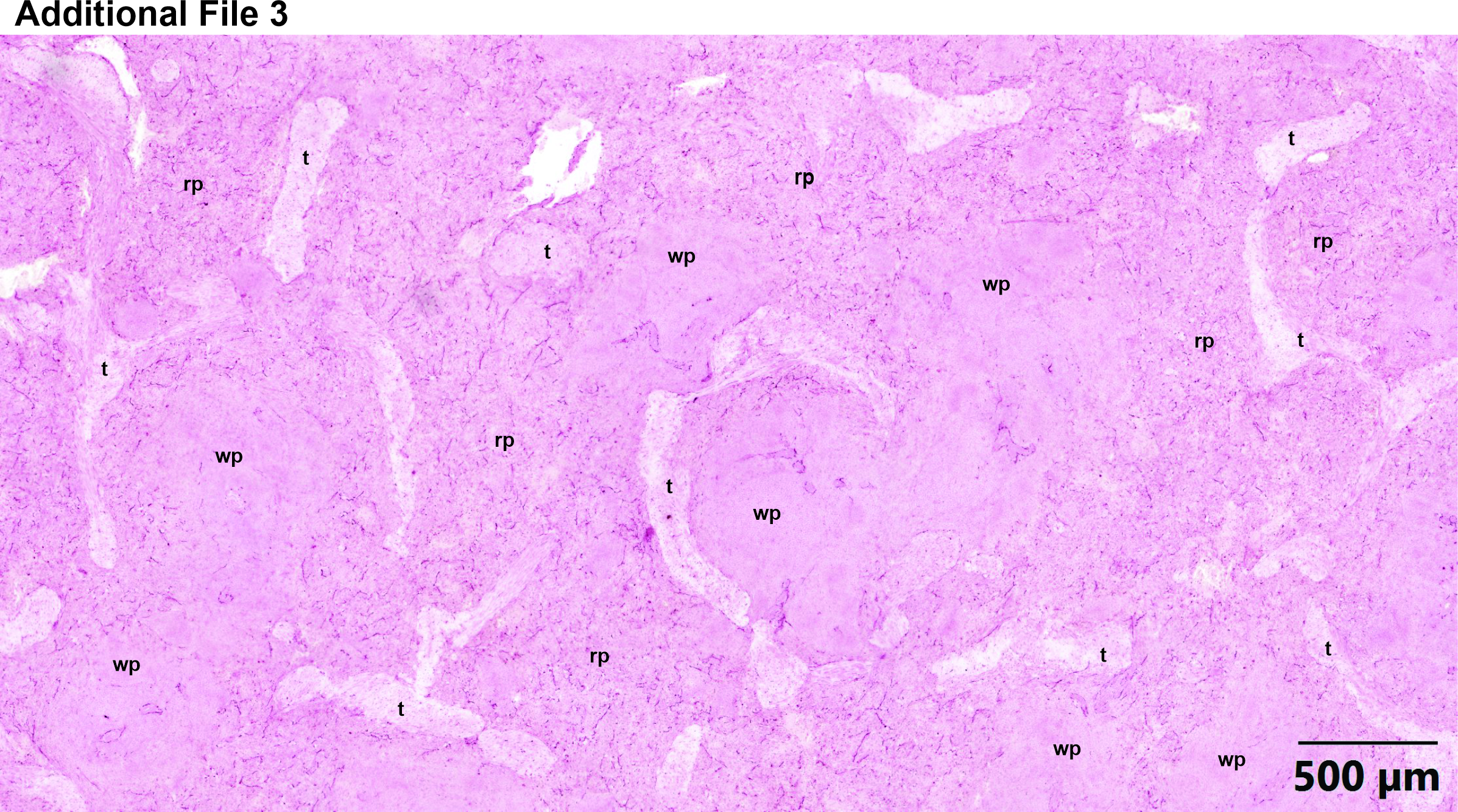

Supplement: Supplementary file 3 — Additional file 3. Montage image showing a large section of porcine spleen stained for NPY. NPY + nerve fibers were found in all regions of pig spleen, with red pulp being the most densely innervated. White pulp (wp), red pulp (rp), and trabeculae (t) are labeled. Montage was created by stitching multiple 10X images using an Olympus BX41 microscope equipped with an Olympus DP74 digital camera and cellSens Dimension software. [file 42234_2022_102_MOESM3_ESM.jpg]

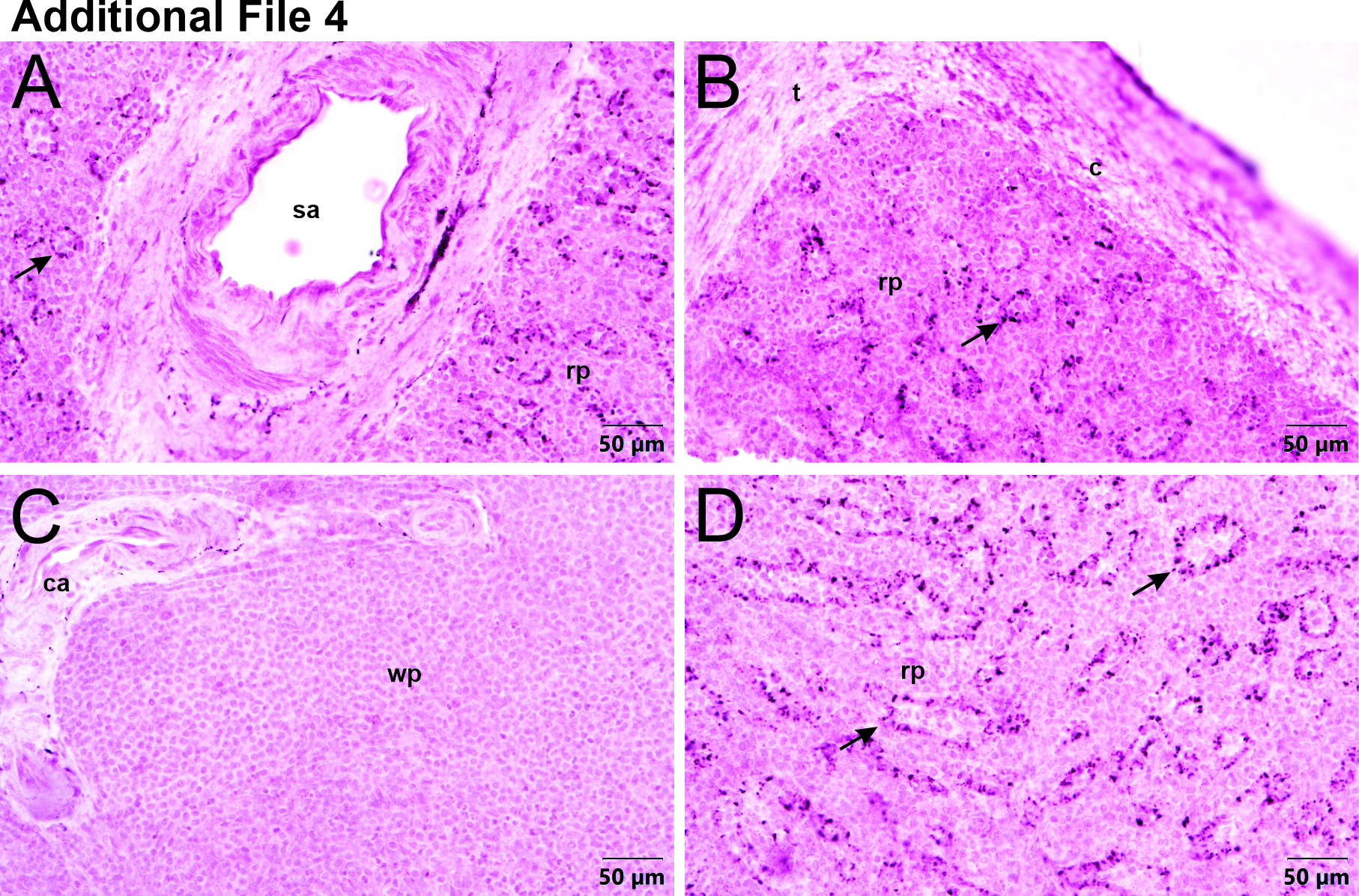

Supplement: Supplementary file 4 — Additional file 4. Spleen from donor 003 exhibited a unique, robust pattern of NPY staining. (A) Image showing NPY nerve staining around a splenic artery (sa) and staining within surrounding red pulp (rp). Arrow indicates NPY staining of littoral cells. (B) Image showing NPY staining of littoral cells (arrow) in the subcapsular region. Note that the trabeculae (t) and capsule (c) are largely free from NPY staining while the red pulp shows dense staining. (C) Image showing lack of innervation within the white pulp (wp) and limited innervation around a central arteriole (ca.). (D) Image showing intense and abundant staining of littoral cells (arrows) within the red pulp. [file 42234_2022_102_MOESM4_ESM.jpg]
